# Retinal Nerve Fiber Layer Decrease during Glycemic Control in Type 2 Diabetes

**DOI:** 10.1155/2010/569215

**Published:** 2010-08-08

**Authors:** Masahiko Sugimoto, Mikio Sasoh, Masashi Ido, Chisato Narushima, Yukitaka Uji

**Affiliations:** ^1^Department of Ophthalmology, School of Medicine, Mie University, 2-174, Edobashi, Tsu, Mie 514-8507, Japan; ^2^Department of Ophthalmology, Yamada Red Cross Hospital, 810, Takamuku, Misono, Ise, Mie 516-0805, Japan

## Abstract

*Purpose*. To assess an effect of glycemic control on retinal nerve fiber layer (RNFL) in type 2 diabetes mellitus. *Methods*. Thirty-eight eyes of 38 patients with type 2 diabetes undergoing blood glucose regulation were enrolled. All patients were examined at (1) initial visit, (2) 1 month, (3) 2 months, and (4) 4-month after the initial examination. On each occasion, glycosylated hemoglobin (HbA1c) levels and optical coherence tomography (OCT) scanning for RNFL thickness were evaluated. 360 degree circular OCT scans with a diameter of 3.4 mm centered on the optic disc were performed. *Results*. Significant RNFL decrease was seen in the superior area between initial and 4 months examination (*P* = .043). The relationship between the changes in HbA1c and the changes in RNFL thickness was observed in superior, temporal, and inferior area (*P* < .05) at 4 months. *Conclusions*. This study suggests that the glycemic control affects RNFL within 4 months.

## 1. Introduction

Diabetes mellitus (DM) is a chronic illness and diabetic retinopathy (DR) is a main cause of blindness in working age [1]. Large randomized-clinical trials suggest that intensive management of hyperglycemia is associated with decreased rates of development and progression of DR in type 1 DM patients [2] and that glycemic control in type 2 DM patients resulted in a 25% risk reduction in retinal microvascular events [3]. Thus, excess glucose is generally considered to be the primary culprit in the progression of DR, and glycemic control can prevent this. But contrary, intensive glycemic control sometimes causes worsening of DR [4]. Glycemic control may have a negative effect on the retina, even though longitude control works well. Not much has been done to clarify what occurs in the retina during glycemic control [5–7], so further studies are needed to elucidate this question.

In glaucoma eyes, retinal nerve fiber layer (RNFL) decreases as a progression [8] and many studies also reported RNFL changes in DM patients [9–14], but to our knowledge, only a few studies have evaluated the relationship between RNFL thickness and glycemic control [15]. These days, various instruments have been established for evaluation of RNFL with no invasion. Especially, optical coherence tomography (OCT) has been proposed as a powerful tool for retinal measurement and provides detailed information with a high resolution [16–18]. OCT can also evaluate RNFL thickness with a high reliability [19]. We hypothesize that RNFL thickness is affected by glycemic control and indicates retinal damage. Here, we conducted a study to assess the effect of glycemic control on RNFL thickness using this useful OCT and to identify what occurs in the retina during glycemic control.

## 2. Subjects and Methods

### 2.1. Subjects

Patients with type 2 DM who had been referred to our endocrinology department for blood glucose regulation were assessed for the study. All patients were Japanese and one eye of each subject was selected randomly for examination. Informed consent was obtained from each of the participants. All subjects were treated in accordance with the declaration of Helsinki for Clinical Research and the study was conducted with the understanding and the consent of the human subject. They were receiving treatment with diet and exercise, oral hypoglycemic agents, or insulin. All subjects were examined as follows: (1) initial visit, (2) 1 month, (3) 2 months, and (4) 4 months after the initial examination. On each occasion, glycosylated hemoglobin (HbA1c) levels and general ophthalmic evaluations including OCT scanning were examined. Fundus examination and OCT scanning were done under dilated condition using 1% tropicamide and 2.5% phenylephrine hydrochloride. Patients underwent fundus examination using a +90 diopter lens and were classified by an independent grader according to the criteria of the ETDRS protocol [20]. The progression of DR was defined as the examination of retinal signs from this classification.

Eligible subjects had to meet the following criteria: (1) subjects whose HbA1c on initial examination was over 7.0, (2) subjects who could be followed up over 4 months, and (3) improved glycemic control evaluated using HbA1c was satisfactorily achieved. A reduction of HbA1c after 4 months by 10% or more was defined as an improvement. HbA1c was measured each time at our hospital in order to provide uniformity among laboratory results. 

Eyes with high myopia, neural ophthalmic anomalies, advanced cataract, cloudy media, and intraocular pressure over 21 mmHg as determined by Goldmann applanation tonometry were excluded from the evaluation. They had no other history of other eye disease, surgery, and photocoagulation. All subjects had normal optic disc appearance. Normal optic disc appearance was defined as a vertical asymmetry of 0.2 or less, a cup-disc ratio of 0.6 or less, and an intact neuroretinal rim without peripapillary hemorrhages, notches, localized pallor, or RNFL defect on clinical examination. Those eyes with progression of DR which required photocoagulation or other therapy during follow-up terms were excluded and therapies were initiated.

### 2.2. OCT Scanning and Analysis

OCT (OCT 1, Zeiss-Humphrey Systems, Dublin, CA) scanning was performed using near-infrared, low-coherence illumination (840 nm) with a tissue resolution of approximately 10–17 *μ*m [15–18]. At each setting, two or three OCT scans were acquired for each patient. From the acquired scans, the best quality, properly aligned scan was chosen for analysis. One trained ophthalmologists (MS) performed all of the procedures.

**Figure 1:**
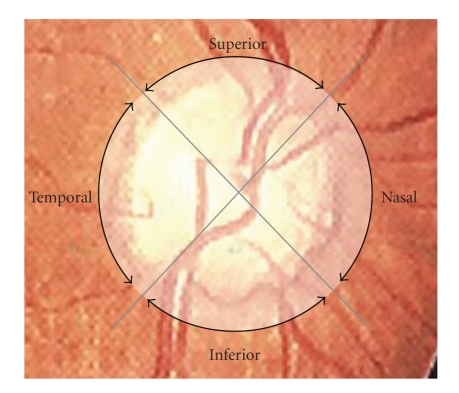
Schematic image of OCT scanning for retinal nerve fiber layer (RNFL) thickness measurements. The circle shows a 360° circular scan with 3.4 mm diameter. Two lines divide the circle into 4 quadrants (temporal, superior, nasal, inferior).

The 360° circular scans with a diameter of 3.4 mm centered on the optic disc were performed as previously described [21, 22]. The scans were centered on the optic disc while patients looked upon landmark points. Fixation was monitored and controlled by the video image of the central optic disc. RNFL thickness was defined as the number of pixels between the anterior and posterior edge of the RNFL detected using the attached automatic boundary detection software (version A5, Humphrey-Zeiss). Each resulting image consists of RNFL thickness measurements at 100 points along a 360° circular ring around the optic disc. RNFL thickness was calculated automatically with existing software: mean (all 360° measurements), temporal quadrant (316°–45° unit circle), superior quadrant (46°–135°), nasal quadrant (136°–225°) and inferior quadrant (226°–315°) thickness (Figure 1). 

### 2.3. Statistical Analysis

Results were shown as the mean values ± standard deviations. Statistical analysis was performed using JMP5.01J (SAS Institute Inc, Cary, NC). Significant differences were determined by use of a non-repeated measures analysis of variance (ANOVA) and SNK test as a post hoc test. Pearson correlation analysis was used for correlation according to a linear regression analysis model. The student's *t*-test for paired group was used for analysis. A *P* value of <.05 was considered statistically significant.

## 3. Results

We identified 38 eyes of 38 patients as meeting the criteria for study. Mean age was 60.5 ± 9.05 (yrs). Eight patients were placed on only diet and exercise therapy, 20 patients were treated with oral hypoglycemic agents alone, and 10 patients were treated with insulin alone. Twenty-four eyes had no retinopathy and 14 eyes had nonproliferative diabetic retinopathy. During the study, no eyes showed progression of retinopathy for advanced treatments.

A significant decrease of HbA1c was seen, which indicates fair glycemic control (*P* = .00233, non-repeated measures of ANOVA, Table 1). 

During OCT analysis in all quadrant areas, no significant RNFL change was seen between the initial and 1 or 2 month examination. But a significant decrease was seen in the superior area between the initial and 4 month examination (*P* = .043, non-repeated measures of ANOVA, Table 2). No significant change was found in other areas. 

**Figure 2:**
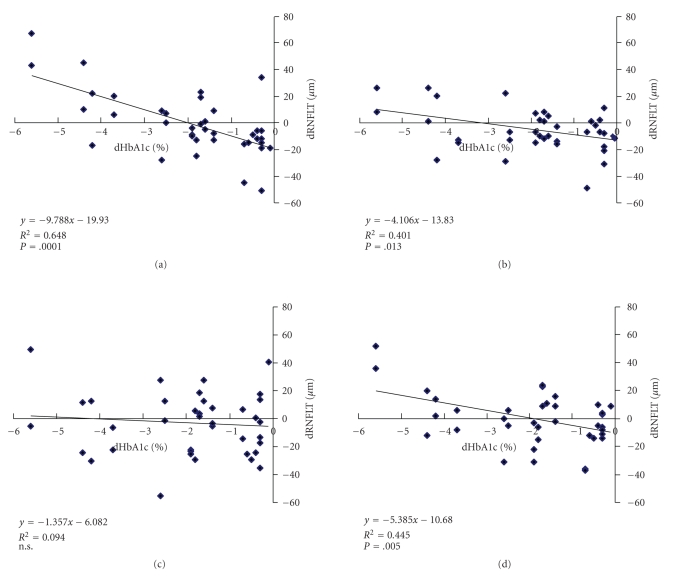
The relationship between HbA1c changes (dHbA1c) and RNFL thickness changes (dRNFLT) in (a) Temporal, (b) Superior, (c) Nasal, (d) Inferior quadrant area. The lines are from a linear regression analysis. There is a significant correlation in temporal (*P* = .0001), superior (*P* = .013) and inferior quadrant area (*P* = .005). There is no significant correlation in nasal quadrant area.

Next, to clarify the relationship between the change in HbA1c (dHbA1c) and the changes in RNFL thickness (dRNFLT) after 4 months of control, we compare them using Pearson correlation analysis. The dRNFLT was related to the dHbA1c in the superior, temporal, and inferior quadrants (*P* < .05 in each area, student's *t*-test, Figure 2). No correlation was seen in the nasal quadrant.

## 4. Discussion

In this study, RNFL thickness decreased in all quadrants area after 4 months of glycemic control. Especially, significant decrease was seen in the superior area. Generally, in glaucomatous eyes, the RNFL is initially damaged in superior area [23–26] and previously we reported that superior RNFL thickness is already decreased in the early stage of DR [22]. Additionally, in vivo study using diabetic animal showed that there were more micro aneurysms seen in superior than in inferior area [27]. So this area is more susceptible to damage than other areas and may have a tendency for higher rates of cell death, which results in RNFL thinning. There remains a question that thinning of the RNFL does not necessarily mean a loss of retinal nerve fibers and the RNFL may have been swollen due to a partial blockade of the axoplasmic flow in the hyperglycemic state. But our data indicates some kind of change, including thinning, occuring in retina components. Thus, superior RNFL thickness is an indicator for retinal damage under the glycemic control. 

According to clinical evidence, there is no doubt that glycemic control is most important for stability of DM and DR. But intensive glycemic control sometimes induces worsening of DR, known as an early worsening (EW) [28]. As the worsening results, hard exudates and macular edema occur. Intensive glycemic control may lead to a transient deterioration of DR and patients who already have background DR are at higher risk for EW changes [29]. On this point, Funatsu et al. emphasized that a 2% or more decrease in HbA1c accelerate DR progression [30]. It should be noted that several reports also have shown interaction between glycemic control and retina change including blood-retina barrier (BRB) or macula edema progression [5–7]. Such vascular breakdown may change the tissue construction and it is reasonable to propose that RNFL damage is followed by this change as an aspect of neurodegeneric change. Some molecular mechanism may participate in this change and some experimental studies have shown that insulin stimulates a cascade of neovascularization. Vascular endothelial growth factor (VEGF) [31, 32] is one of the major mediators of neovascularization or ischemia. Insulin up-regulates VEGF *in vitro* and enhances vascular permeability or proliferative effects [33, 34] which results in proliferative retinal changes. Thus, insulin itself plays an important role in neovascularization or BRB breakdown and may be one of the causes of several complications during glycemic control, including EW. Patients in this study were treated variably; some were given insulin, some were given oral agent, and some were treated without medication. Because of the small number of patients, there was no significant RNFL thickness change for our ten of insulin-treated patients and other patients in each area between the initial visit and each subsequent visit (*P* > .05 in each area, data not shown). And there is a report that progression of DR differs by insulin type [35], so further clarification is needed to examine the relation between RNFL damage and insulin. This is a pilot study which gives support to further studies in these areas. Having had more sample number would have been helpful in truly allowing the hypothesis that the correlation between HbA1c levels and RNFL thinning is due to insulin or other factors. And in addition, there exists several problems in our study. First, the patients are poorly characterized in aspects of several factors such as age, sex, duration of diabetes, coexistent renal disease and blood pressure (BP). As several studies mentioned about importance of BP control for prevention of DR [36], it is important to consider BP control. There is a possibility that increased BP and secondary increase in cerebrospinal fluid pressure may relate with RNFL change. Second, we did not use updated technology of OCT which can detect much detailed change than OCT 1, which we used here. And third, there is a possibility that visual fields evaluation may support our results much precisely. On these points, we need much larger and sufficiently-powered follow-up study.

## 5. Conclusion

In summary, this study suggests that the glycemic control affects RNFL within 4 months and superior RNFL thickness can be used as an indicator for this change. Moreover, it may provide further new information regarding the mechanism of EW.

## Figures and Tables

**Table 1:** Change in HbA1c during glycemic control.

HbA1c (%)	
Initial	9.20 ± 1.53
1M	8.43 ± 1.32*
2M	7.44 ± 1.28*
4M	7.35 ± 1.29*

The HbA1c is expressed in mean ± standard deviations.

*Statistically significant values at *P* < .05.

**Table 2:** Change in retinal nerve fiber layer thickness during glycemic control.

	Mean	Temporal	Superior	Nasal	Inferior	
Initial	127.2 ± 13.8	111.2 ± 16.8	145.7 ± 17.3	110.2 ± 15.8	142.2 ± 17.9	
1M	126.0 ± 12.3	109.4 ± 18.0	143.7 ± 16.8	110.2 ± 17.0	141.3 ± 16.3	
2M	126.7 ± 12.1	111.8 ± 17.5	143.2 ± 18.1	108.8 ± 14.9	143.4 ± 15.0	
4M	124.4 ± 12.7	109.5 ± 18.9	139.9 ± 14.6*	106.8 ± 17.1	141.7 ± 16.9	
						(*μ*m)

RNFL thickness is expressed in mean ± standard deviations.

*Statistically significant values at *P* < .05.
